# Papulonodular mucinosis with features of discoid lupus erythematosus

**DOI:** 10.1016/j.jdcr.2023.12.022

**Published:** 2024-01-20

**Authors:** Emma Hansen, Cory Pettit, Catherine G. Chung, Abraham M. Korman

**Affiliations:** a The Ohio State University College of Medicine, Columbus, Ohio; b Department of Dermatology, The Ohio State University Wexner Medical Center, Columbus, Ohio; c Department of Pathology, The Ohio State University Wexner Medical Center, Columbus, Ohio

**Keywords:** dermatopathology, lupus erythematosus, papulonodular mucinosis, skin of color

## Introduction

Papulonodular mucinosis is a cutaneous dermal subtype that comprises a small percentage of lupus erythematosus (LE) cases, with an estimated 80% of these cases occurring in the setting of systemic LE (SLE).^1^ In a review of the literature, approximately 76% and 20% of papulonodular mucinosis cases were noted to be associated with SLE and discoid LE (DLE), respectively.^2^ Substantial dermal mucin deposition leads to the characteristic clinical and histopathological manifestations, which are usually readily distinguishable from other cutaneous LE (CLE) subtypes.^1^, ^2^, ^3^, ^4^, ^5^, ^6^ We describe a patient with papulonodular mucinosis with concurrent features of DLE.

## Case report

**Fig 1:**
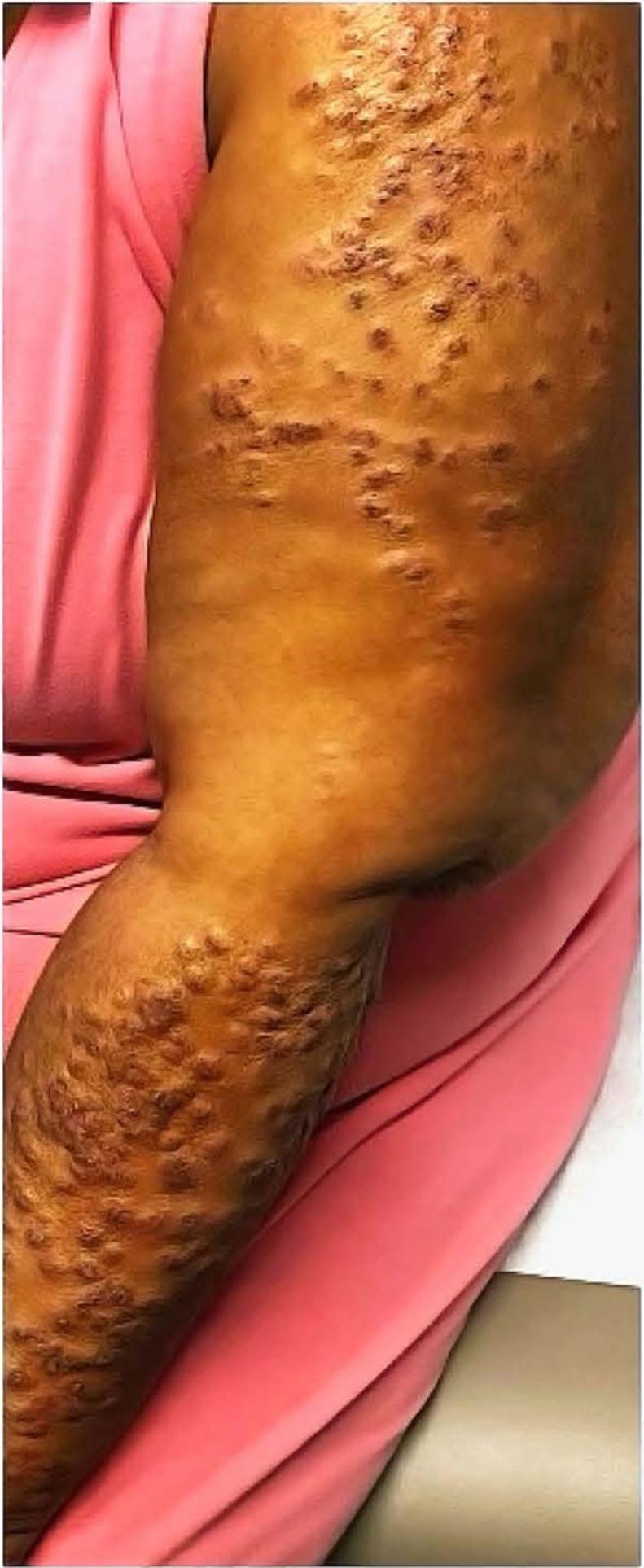
Left arm and forearm with erythematous to violaceous, dome-shaped papules, some with central follicular plugging.

**Fig 2:**
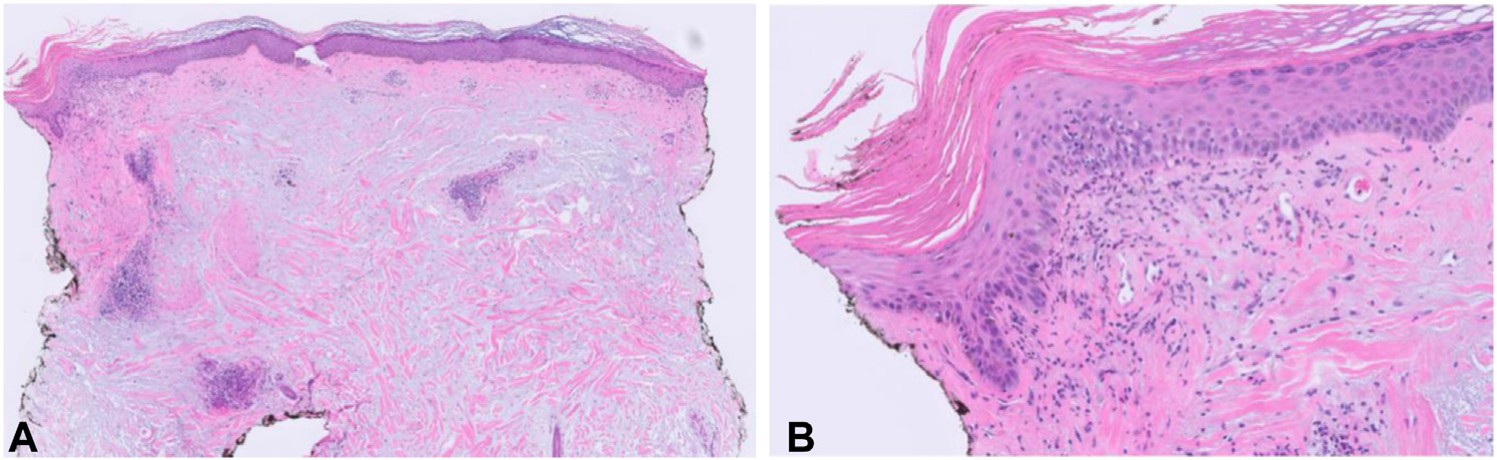
Histopathologic Findings. **A,** Punch biopsy sample stained with hematoxylin and eosin demonstrating extensive dermal mucin with wide separation of collagen fibers; original magnification ×2. There is focal interface change with underlying mononuclear perivascular infiltrate. **B,** Higher magnification of keratinocyte necrosis and a dilated follicular ostium with overlying hyperkeratosis; original magnification ×20.

An African American woman in her 50s presented with an intensely pruritic rash on the arms, legs, and scalp. Two and a half years prior, she had been diagnosed with rheumatoid arthritis and Sjogren’s syndrome with keratoconjunctivitis sicca. Her symptoms improved on leflunomide after being unable to tolerate methotrexate. Pertinent laboratory results included positive antinuclear antibody (>1:160) and anti-Sjogren’s-syndrome-related antigen A-60 antibody, negative anti-double-stranded deoxyribonucleic acid, anti-Smith, anti-ribonucleoprotein, anti-Sjogren’s-syndrome-related antigen A-52 and anti-Sjogren’s-syndrome-related-antigen B antibodies, nonreactive syphilis antibody, and normal thyroid stimulating hormone and free Thyroxine, serum immunofixation, urinalysis, and complete blood count. Physical exam revealed well-delineated, dome-shaped papules in a photodistributed pattern (Fig 1). The papules were erythematous to violaceous in color, and some had focal follicular plugging. Punch biopsy demonstrated extensive mucin deposition in the dermis with wide separation of collagen fibers. Additional findings included focal vacuolar interface change, follicular plugging, and focal periadnexal and perivascular lymphocytes (Fig 2). Hydroxychloroquine (200 mg twice daily) and topical steroids were prescribed, but her symptoms persisted at 3-month follow-up. After adding mycophenolate mofetil (500 mg twice daily) and a 3-week prednisone taper (starting at 60 mg daily), her rash resolved.

## Discussion

Although papulonodular mucinosis is associated with SLE in the majority of cases, it can also occur in the absence of systemic findings.^1^, ^2^, ^3^, ^4^, ^5^, ^6^ The cutaneous manifestations of papulonodular mucinosis include raised papules and nodules localized to the upper body and trunk that are often inconspicuous, but can appear erythematous or violaceous, particularly in patients with skin of color.^1^, ^2^, ^3^, ^4^, ^5^, ^6^ The pathological hallmark of papulonodular mucinosis is significant mucin throughout the dermis, and epidermal change including interface dermatitis tends to be absent.^1^, ^2^, ^3^, ^4^, ^5^, ^6^ Similar histopathological findings are observed in other dermal variants of CLE, including Jessner-Kanof lymphocytic infiltration of the skin and tumid lupus.^4^^,^^7^ However, in these entities, characteristic features include prominent perivascular lymphocytic infiltrate throughout the papillary and reticular dermis and interstitial dermal mucinosis, which may require special stains to appreciate.^4^^,^^7^ The quantity of mucin in papulonodular mucinosis is far more substantial.

In contrast, DLE, a dermoepidermal subtype of CLE, presents with well-demarcated plaques with prominent epidermal changes including dyspigmentation and scale.^4^ Keratinocyte apoptosis, epidermal atrophy, basement membrane thickening, follicular plugging, and periadnexal and perivascular inflammatory cells are characteristic pathological features of DLE.^4^ While dermal mucin may be observed in DLE, the degree of mucin is typically not significant enough to result in palpable cutaneous manifestations.^4^

The combination of massive, pooling dermal mucin between collagen fibers and extensive, photodistributed dermally based papules was most consistent with papulonodular mucinosis, occurring in the absence of SLE. Overall, our patient’s clinical picture can be contrasted with DLE, as her cutaneous findings appeared as papules rather than plaques, and we did not observe pronounced hyperkeratosis, scarring, or pigment changes characteristic of DLE.^4^ However, many of the papules were characterized by central follicular scale, correlating with interface change, perivascular and periadnexal lymphocytic infiltrate, and follicular hyperkeratosis on histopathology, which are features associated with DLE rather than papulonodular mucinosis.^4^ Therefore, we believe this case is best characterized as an unusual variant of papulonodular mucinosis with subtle overlapping clinical and histopathologic features of DLE.

Therapeutic options for papulonodular mucinosis include hydroxychloroquine, glucocorticoids, topical calcineurin inhibitors, methotrexate, mycophenolate mofetil, and thalidomide.^1^^,^^2^^,^^4^, ^5^, ^6^^,^^8^

We describe a patient presenting with overlapping features of papulonodular mucinosis, Jessner-Kanof lymphocytic infiltrate, and DLE, highlighting their clinical and histopathological continuum within the spectrum of chronic CLE. In addition, we uniquely highlight the appearance of papulonodular mucinosis in a patient with skin of color, which can be variable and possibly more difficult to recognize than on white skin. It is critical to report such cases to facilitate greater recognition of papulonodular mucinosis in a variety of skin types.

## Conflicts of interest

Drs Chung and Korman are members of the JAAD Case Reports Editorial Board. Emma Hansen and Cory Pettit have no additional conflicts of interest to declare.
